# Supplementary material for: Public-private partnership to rapidly strengthen and scale COVID-19 response in Western Kenya
Source: Front Public Health. 2023 Jan 17;10:837215. doi: 10.3389/fpubh.2022.837215 (PMC9887331; doi:10.3389/fpubh.2022.837215)
Supplement: Supplementary file 1 [file Data_Sheet_1.PDF]

MINISTRY OF HEALTH
Division of Disease Surveillance and Response

Case investigation form for 2019 Novel Coronavirus (2019-nCoV)

Date of reporting / Entering data: [_D_][_D_]/[_M_][_M_]/[_Y_][_Y_][_Y_][_Y_]

Reporting Health Facility:/ Facility of testing ______________________________

Sub Country:______________________________Country:______________________________

Detected at point of entry □ No □ Yes □ Unknown If yes, date [_D_][_D_]/[_M_][_M_]/[_Y_][_Y_][_Y_][_Y_]

Section 1: Patient information

Unique Case Identifier (Case ID): ____________________________

Age in years: [__][__][__] if < 1 year old, [_][_] in months or if < 1 month, [_][_] in days

Sex at birth: □ Male □ Female □ Unknown

Race □ African □ Indian □ Caucasian □ Mixed race □ Other

Phone number _____________

Name _____________

Occupation

Place where the case was diagnosed: Health Facility: __________________

County of residence:_____________ Sub County of residence: __________

Ward of residence: (free text)____________________ Residence (Village/estate): (free text)________________

Nearest Health facility (Free text)____________________

Nearest landmark ____(Free text)________________

Patient usual place of residency: Country: ______________________

Admin Level 1 (province):______________________Admin Level 2 (district): ____________________________

Section 2: Clinical information

Patient clinical course

Patient symptoms (check all reported symptoms):

□ History of fever / chills
□ General weakness
□ Cough
□ Sore throat
Runny nose
□ Asymptomatic
□ Other, specify ____

□ Shortness of breath/difficulty brathing
□ Diarrhoea
□ Nausea/vomiting
□ Headache
□ Irritability/Confusion

□ Pain (check all that apply)
( ) Muscular ( ) Chest
( ) Abdominal ( ) Joint

□ Loss of sense of taste □ Loss of sense of smell

Patient signs:

Temperature: [_][_][_] □°C / □ F

Check all observed signs:

□ Pharyngeal exudate
□ Conjunctival injection
□ Seizure
□ Coma
□ Dyspnea / tachypnea
□ Abnormal lung auscultation
□ Abnormal lung X-Ray findings
□ Other, specify: ____

Underlying conditions and comorbidity (check all that apply)**:**

□ Pregnancy (trimester:__________) □ Post-partum (< 6 weeks) □ Hypertension

□ Cardiovascular disease, including hypertension □ Immunodeficiency, including HIV

□ Diabetes Mellitus
□ Renal disease
□ Liver disease
□ Chronic lung disease
□ Chronic neurological or neuromuscular disease
□ Malignancy
□ Other, specify: ______________________________

Date of onset of symptoms: [_D_][_D_]/[_M_][_M_]/[_Y_][_Y_][_Y_][_Y_

Admission to hospital:/ Is the person admitted? □ No □ Yes □ Unknown

Facility of admission ___________________________

date of admission to hospital:[_D_][_D_]/[_M_][_M_]/[_Y_][_Y_][_Y_][_Y_]

Date of isolation:[_D_][_D_]/[_M_][_M_]/[_Y_][_Y_][_Y_][_Y_] Was the patient ventilated: □ No □ Yes □ Unknown

Health status (circle) at time of reporting: Stable / Severely ill / dead / unknown

Date of death, if applicable: [_D_][_D_]/[_M_][_M_]/[_Y_][_Y_][_Y_][_Y_]

□

Section 3: Exposure and travel information in the 14 days prior to symptom onset (prior to reporting if asymptomatic)

Has the patient **travelled** in the 14 days prior to symptom onset? □ No □ Yes □ Unknown

If yes, please specify the places the patient travelled:

	Country	City
1.	______________________________	______________________________
2.	______________________________	______________________________

Has the patient **visited any health care facility (ies)** in the 14 days prior to symptom onset? □ No □ Yes □ Unknown

Has the patient had **close contact**[1] with a person with acute respiratory infection in the 14 days prior to symptom onset?

□ No □ Yes □ Unknown

If yes, contact setting (check all that apply):

□ Health care setting □ Family setting □ Work place □ Unknown □ Other, specify: ______________________

Has the patient **had contact with a probable or confirmed case** in the 14 days prior to symptom onset? **:**

□ No □ Yes □ Unknown

If yes, please list unique case identifiers of all probable or confirmed cases:

Case 1 identifier.____________________Case 2 identifier.______________________Case 3 identifier. ________________________

If yes, contact setting (check all that apply):

□ Health care setting □ Family setting □ Work place □ Unknown □ Other, specify: ______________________

If yes, location/city/country for exposure: _____________________

Have you visited any **live animal markets** in the 14 days prior to symptom onset? □ No □ Yes □ Unknown

If yes, location/city/country for exposure: _____________________

Vaccinated for Covid___□ Yes □ No □ Unknown

If Yes date of vaccination :[_D_][_D_]/[_M_][_M_]/[_Y_][_Y_][_Y_][_Y_]

Section 4: Laboratory Information

RDT testing? ☐ 1=Yes 2=No

a. Type of antigen Test used? ☐ Bionote ☐ Abbott ☐ SD Biosensor ☐ Other

b. RDT Test result ☐ positive ☐ negative ☐ indeterminate ☐ Not Applicable

PCR testing? ☐ 1=Yes 2=No

c. PCR Test result ☐ positive ☐ negative ☐ indeterminate ☐ Not Applicable

d. Date of specimen collection: [_D_][_D_]/[_M_][_M_]/[_Y_][_Y_][_Y_][_Y_]
e. Specimen type: ☐ NP Swab ☐ OP Swab ☐ Serum ☐ Sputum ☐ Tracheal Aspirate
Other (specify): ___________________________________
f. Date specimen sent to the lab: [_D_][_D_]/[_M_][_M_]/[_Y_][_Y_][_Y_][_Y_]

(To be completed by the confirming lab)

g. Name of confirming lab: ____
h. Please specify which assay was used:____________________________Sequencing done?: ◻ Yes ◻ No ◻ Unknown
i. Preliminary lab PCR result: _______________________ PCR cycle threshold value: _______________
j. Date of laboratory confirmation: [_D_][_D_]/[_M_][_M_]/[_Y_][_Y_][_Y_][_Y_]

[1] Close contact' is defined as: 1. Health care associated exposure, including providing direct care for nCoV patients, working with health care workers infected with novel coronavirus, visiting patients or staying in the same close environment of a nCoV patient. 2. Working together in close proximity or sharing the same classroom environment with a with nCoV patient. 3. Traveling together with nCoV patient in any kind of conveyance. 4. Living in the same household as CoV patient
