# Supplementary material for: Public-private partnership to rapidly strengthen and scale COVID-19 response in Western Kenya
Source: Front Public Health. 2023 Jan 17;10:837215. doi: 10.3389/fpubh.2022.837215 (PMC9887331; doi:10.3389/fpubh.2022.837215)
Supplement: Supplementary file 2 [file Data_Sheet_2.PDF]

Supplementary materials

Figure 1. Screenshots Operational Dashboard COVID-Dx

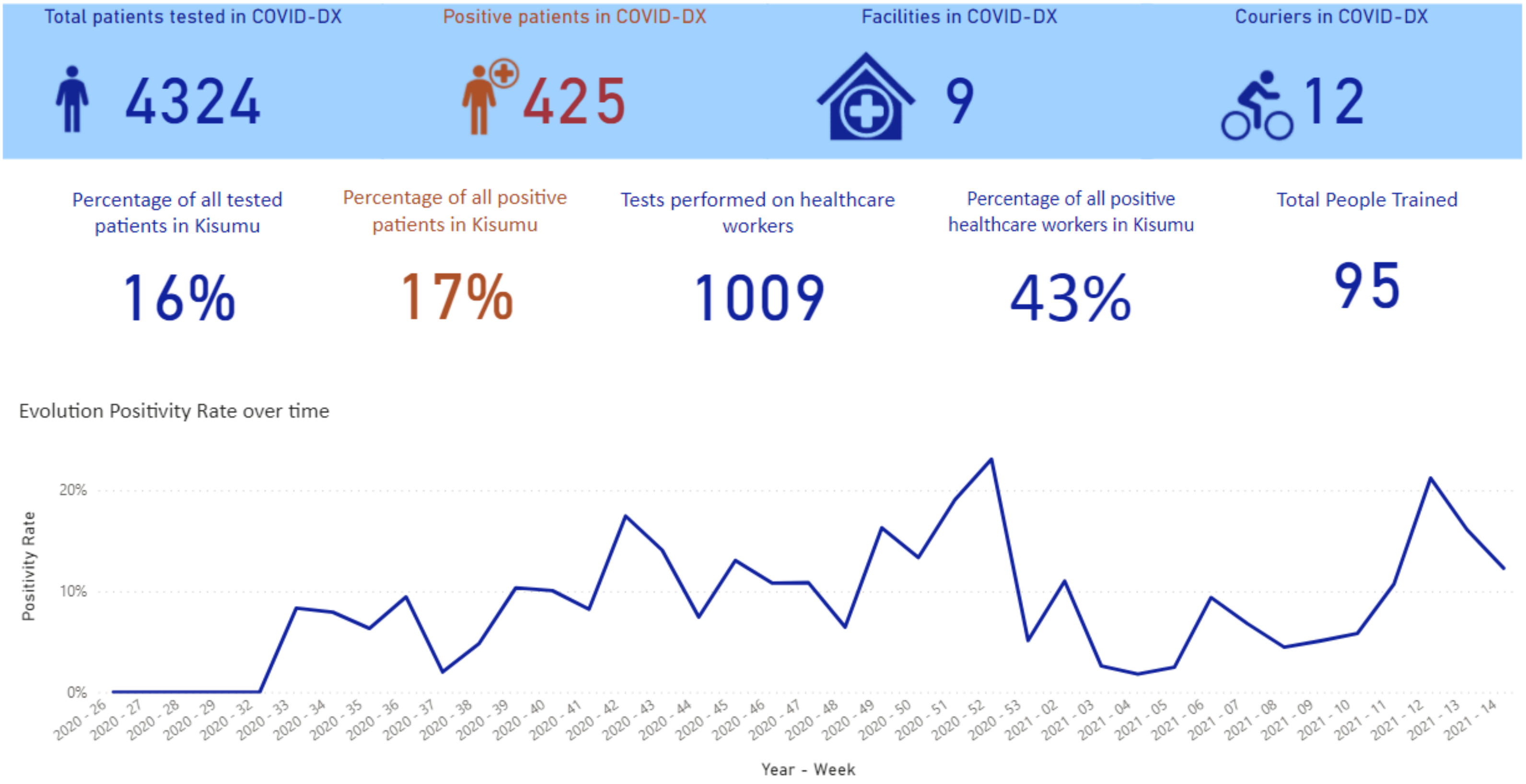

Testing results

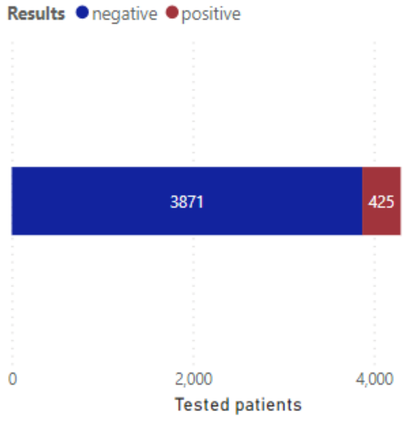

Total patients tested grouped by results.

Patients enrolled in COVID-DX per day

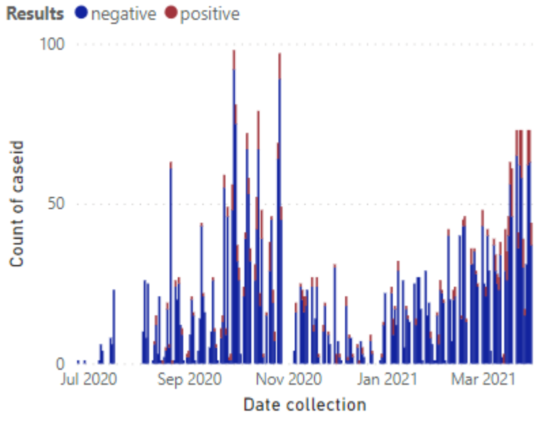

Overview of the total number of patients enrolled over time.

Time between sample collection and data entry completion

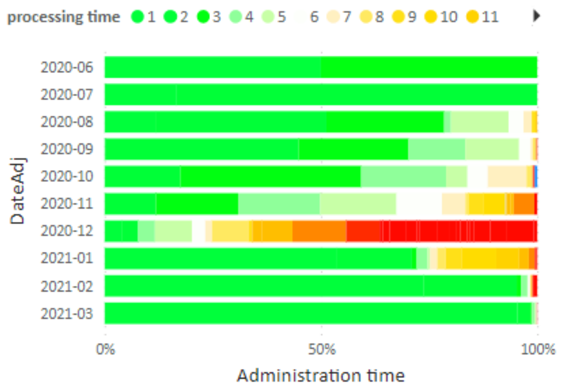

Number of days between sample collection and results reporting

Positive patients reported asymptomatic

23%

Frequency with which symptoms have been reported in positive cases

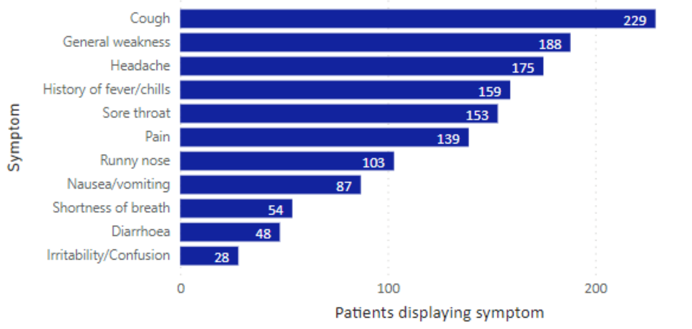

In the graph above, a patient can be represented multiple times if (s)he displayed multiple symptoms.

Underlying conditions (when applicable)

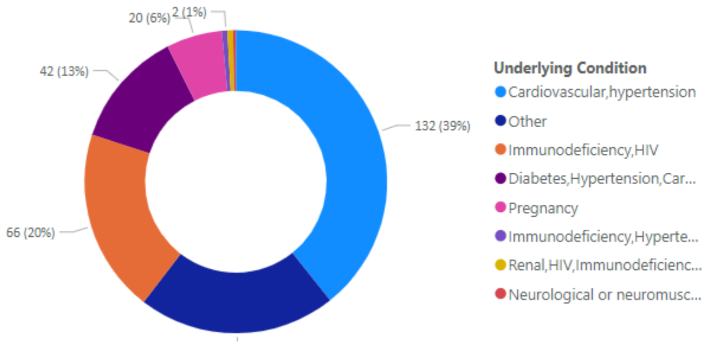

Supplementary Materials Manuscript “Public-private partnership to strengthening COVID-19 response in Kisumu, Kenya”

Figure 2. COVID-Dx roll-out in the context of Kenyan COVID pandemic highlights

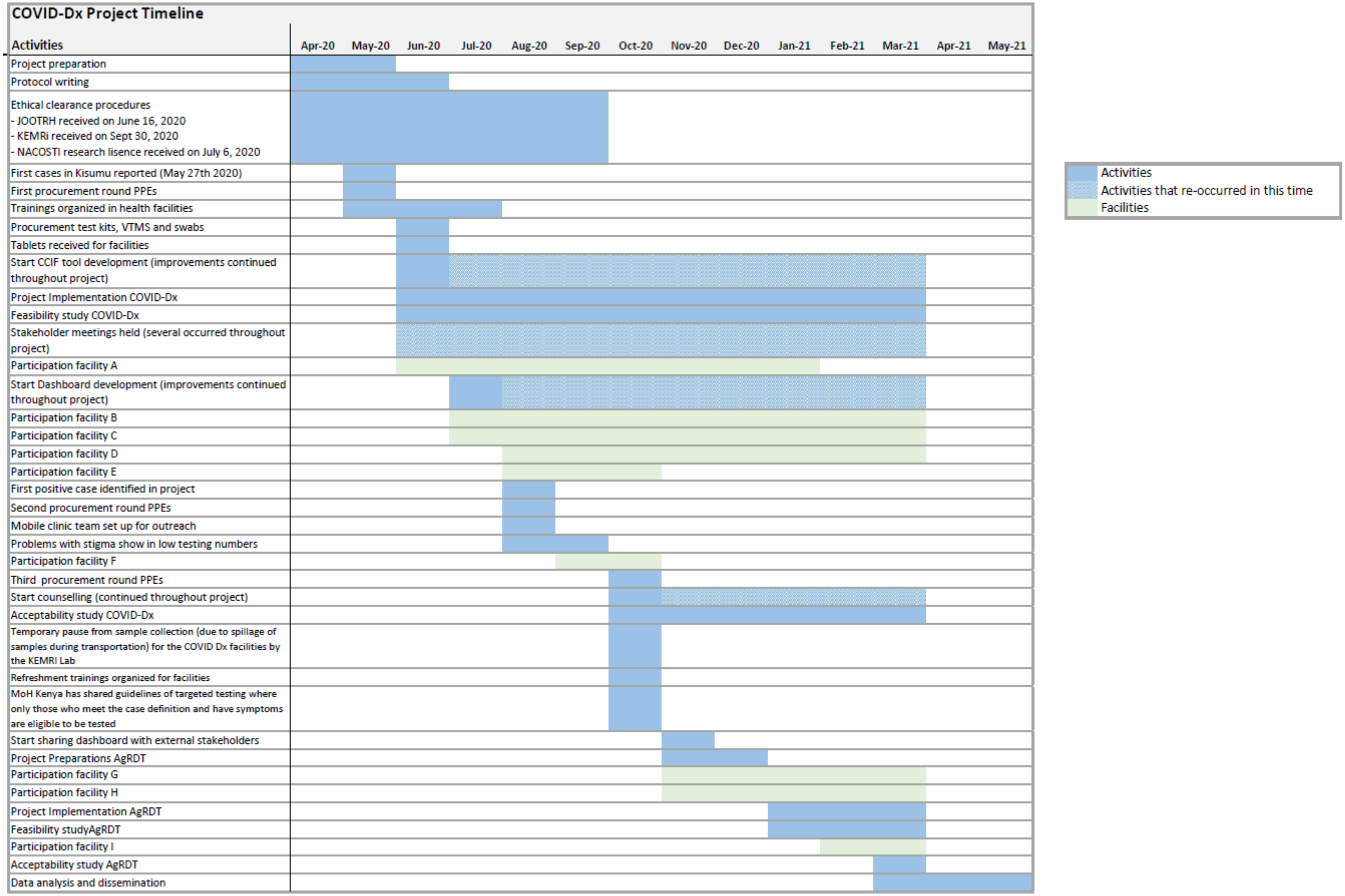

Supplementary Materials Manuscript “Public-private partnership to strengthening COVID-19 response in Kisumu, Kenya”
